# Prediction of Delivery Within 7 Days After Diagnosis of Early Onset Preeclampsia Using Machine-Learning Models

**DOI:** 10.3389/fcvm.2022.910701

**Published:** 2022-07-01

**Authors:** Cecilia Villalaín, Ignacio Herraiz, Paula Domínguez-Del Olmo, Pablo Angulo, José Luis Ayala, Alberto Galindo

**Affiliations:** ^1^Fetal Medicine Unit, Department of Obstetrics and Gynecology, University Hospital “12 de Octubre”, Research Institute Hospital 12 de Octubre (imas12), Primary Care Interventions to Prevent Maternal and Child Chronic Diseases of Perinatal and Developmental Origin (RICORS Network), Complutense University of Madrid, Madrid, Spain; ^2^Department of Computer Architecture and Automation, Faculty of Informatics of the Complutense University, Madrid, Spain

**Keywords:** preeclampsia, prediction, machine-learning, HELLP syndrome, placental abruption

## Abstract

**Background:**

Early onset preeclampsia (eoPE) is a hypertensive disorder of pregnancy with endothelial dysfunction manifested before 34 weeks where expectant management is usually attempted. However, the timing of hospitalization, corticosteroids, and delivery remain a challenge. We aim to develop a prediction model using machine-learning tools for the need for delivery within 7 days of diagnosis (model D) and the risk of developing hemolysis, elevated liver enzymes, and low platelets (HELLP) syndrome or *abruptio placentae* (model HA).

**Materials and Methods:**

A retrospective cohort of singleton pregnancies with eoPE and attempted expectant management between 2014 and 2020. A Mono-objective Genetic Algorithm based on supervised classification models was implemented to develop D and HA models. Maternal basal characteristics and data gathered during eoPE diagnosis: gestational age, blood pressure, platelets, creatinine, transaminases, angiogenesis biomarkers (soluble fms-like tyrosine kinase-1, placental growth factor), and ultrasound data were pooled for analysis. The most relevant variables were selected by bio-inspired algorithms. We developed basal models that solely included demographic characteristics of the patient (D1, HA1), and advanced models adding information available at diagnosis of eoPE (D2, HA2).

**Results:**

We evaluated 215 eoPE cases and 47.9% required delivery within 7 days. The median time-to-delivery was 8 days. Basal models were better predicted by K-nearest-neighbor in D1, which had a diagnostic precision of 0.68 ± 0.09, with 63.6% sensitivity (Sn), 71.4% specificity (Sp), 70% positive predictive value (PPV), and 65.2% negative predictive value (NPV) using 13 variables and HA1 of 0.77 ± 0.09, 60.4% Sn, 80% Sp, 50% PPV, and 87.9% NPV. Models at diagnosis were better developed by support vector machine (SVM) using 18 variables, where D2’s precision improved to 0.79 ± 0.05 with 77.3% Sn, 80.1% Sp, 81.5% PPV, and 76.2% NPV, and HA2 had a precision of 0.79 ± 0.08 with 66.7% Sn, 82.8% Sp, 51.6% PPV, and 90.3% NPV.

**Conclusion:**

At the time of diagnosis of eoPE, SVM with evolutionary feature selection process provides good predictive information of the need for delivery within 7 days and development of HELLP/*abruptio placentae*, using maternal characteristics and markers that can be obtained routinely. This information could be of value when assessing hospitalization and timing of antenatal corticosteroid administration.

## Introduction

Preeclampsia (PE) is a multisystem disorder of pregnancy defined as *de novo* or worsening hypertension from 20 weeks of gestation with endothelial dysfunction manifested as proteinuria or end-organ damage (1). Its associated complications include refractory hypertension, renal failure, eclampsia, stroke, pulmonary edema, hemolysis, elevated liver enzymes, and low platelets (HELLP) syndrome or *abruptio placentae*, making PE a leading cause of maternal morbidity and mortality, with 50,000 maternal deaths yearly worldwide, most of them in developing countries (2). Unfortunately, there is still no treatment for the disease beyond timely delivery (3).

The early onset PE (eoPE) subtype, defined as that diagnosed before 34 + 0 weeks, is a critical challenge since prompt delivery exposes the fetus to the consequences of prematurity. Therefore, expectant management is usually attempted at the expense of exposing the mother to the risk of developing complications (4). In eoPE, abnormal trophoblastic invasion in early pregnancy causes placental hypoperfusion and hypoxia, which compromise maternal–fetal exchange of nutrients and oxygen. As a response, an excess of placental antiangiogenic factors, such as soluble fms-like tyrosine kinase-1 (sFlt-1), are released into the maternal circulation. This reduces the bioavailability of proangiogenic factors, such as the placental growth factor (PlGF). The angiogenic imbalance induces maternal endothelial dysfunction, being responsible for hypertension, proteinuria, and end-organ disease (5). The increase of the sFlt-1/PlGF ratio is related to eoPE (6), being detectable up to 5 weeks before clinical symptoms (7). Moreover, there is an inverse relationship between the value of the sFlt-1/PlGF ratio and the time lapse until it is necessary to deliver due to disease progression. In particular, extremely high values of the ratio have been related to complications, such as HELLP syndrome and *abruptio placentae* (8), which are otherwise difficult to anticipate. Therefore, the integration of these biomarkers with other clinical, analytical, and ultrasound tools could be of use in the prediction of eoPE progression after diagnosis.

Current predictive models of PE have several limitations such as their short-term (within 48 h) predictive capability, the prediction of maternal but not fetal complications, the inclusion of solely severe cases from onset (9), or the focus on the development of PE but not its complications (10). Others have only included data obtained in the first trimester, and most of them have not used angiogenesis biomarkers (9, 11). Approaching such complex optimization problems can be challenging and supervised machine-learning techniques, which generate classification models that analyze patterns and trends in the variables of a large volume of data to ultimately predict the course and progression of the disease, can be of use (12). Our aim is to develop two predictive models with available data at the time of diagnosis of eoPE: (1) need to deliver within 7 days and (2) risk of developing HELLP syndrome or *abruptio placentae*.

## Materials and Methods

Our retrospective cohort study was on singleton women with a diagnosis of eoPE between January 2014 and December 2020. Inclusion criteria were singleton pregnancies with a diagnosis of PE before 34 + 0 weeks and attempted expectant management. Cases with congenital anomalies, lack of angiogenesis biomarkers determination at diagnosis (±48 h), or loss to follow-up were excluded. The study was approved by the local Ethics Committee (n 21/113). Due to its retrospective, non-interventional nature with the use of de-identified information, the requirement of informed consent was waived.

### Data Collection, Follow-Up, and Outcome Measures

#### Baseline Characteristics

Maternal characteristics include age, height, weight, smoking status, race, method of conception, low-dose aspirin intake, heparin prophylaxis, and risk factors for PE and other PD-related disorders according to the National Institute for Health and Care Excellence (NICE) guidelines (13) were collected from the medical records. During the study period, PE was screened according to the NICE risk-factor guidelines criteria. Those women with ≥ 1 high risk factor or ≥ 2 moderate ones were considered at high risk of PE, and a recommendation of prophylaxis with aspirin was made. However, not all women were evaluated at first in our center so the screening protocol may have differed. Furthermore, there were some women with low molecular heparin treatment, either in the context of thrombophilia, systemic erythematosus lupus, or assisted reproduction techniques. Uterine artery evaluation at 20 weeks was recorded and centiles were calculated (14). Gestational age (GA) was estimated according to the American College of Obstetricians and Gynecologists, that is, reliable last menstrual period was corrected by the crown-rump length before 14 + 0 weeks or biparietal diameter from 14 + 0 weeks to 21 + 6 weeks, when a significant discrepancy of more than 7 days or more than 10 days was found, respectively (15).

#### Preeclampsia Diagnosis and Management

Preeclampsia was defined as the presence of both hypertension and proteinuria, according to the National High Blood Pressure Education Program Working Group on High Blood Pressure in Pregnancy (16). In the clinical setting, patients were managed as having PE even in the absence of proteinuria when other severity criteria were met and indications for delivery followed current recommendations (17).

At diagnosis, a complete blood count, biochemistry including angiogenesis biomarkers, and spot protein/creatinine ratio were measured. The sFlt-1 and PlGF concentrations (picograms per milliliter) were determined using an automated assay system (Cobas^®^ 6000 e701 module, Roche Diagnostics, Penzberg, Germany). The sFlt-1/PlGF ratio was expressed in absolute values, and the obstetricians involved were aware of the results of the sFlt-1/PlGF ratio. Values ≤ 38 were considered to rule out PE ≥ 85 as “aid in diagnosis,” and > 655 as a high risk of the need to deliver within 48 h (18). This information was not intently used to indicate delivery, although its knowledge could have influenced the clinicians in the interpretation of the severity of PE-related symptoms that leads to decisions about the continuation of the pregnancy.

Corticoids for fetal maturity (betamethasone 12 mg/day for 2 days) were administered before 34 + 6 weeks if fetal viability was reached [provided that the GA was ≥ 24 + 0 weeks in normally grown fetuses or 26 + 0 weeks and estimated fetal weight (EFW) ≥ 500 g in growth restricted fetuses]. A repeated cycle of corticoids was considered after 1 week of the administration of the first cycle if the GA remained below 34 + 6 weeks. Magnesium sulfate was indicated in the presence of severity features or regardless of maternal status when there was a risk of imminent delivery < 32 weeks.

Expectant management was initially attempted for fetal interest whenever the GA was ≥ 24 weeks and was discussed with the parents at earlier weeks. Severe complications that indicated immediate delivery irrespectively of GA (19) were pulmonary edema, refractory hypertension (uncontrolled blood pressure despite two antihypertensive medications at maximum doses), HELLP syndrome, *abruptio placentae* [defined as placental detachment prior to delivery of the fetus, including the identification of a retroplacental hematoma or evidence of blood clot in at least 20% of its surface based on clinical data provided by the attending obstetrician at delivery (20)], renal failure (oliguria < 500 mL/24 h, creatinine > 1.2 mg/dl), and neurological deficit (persisting visual alterations, stupor, clonus, or eclampsia). Delivery was also recommended after 34 + 0 weeks in the presence of severe features of PE and in any PE case after 37 + 0 weeks.

#### Fetal Assessment

Fetal assessment including a detailed anatomical scan and growth evaluation was undertaken at our placental dysfunction consult within 48 h of eoPE diagnosis. Fetal weight was estimated (21) and centiles customized (22) to maternal and fetal characteristics. Fetal growth restriction (FGR) was diagnosed according to a stage-based classification (23): stage I was considered in cases with normal fetal Doppler or abnormal but with antegrade umbilical artery (UA) flow; stage II was those with absent-end diastolic UA flow; stage III those with reversed end-diastolic UA flow or ductus venosus PI > 95th centile, and finally stage IV was limited to cases with a reversed a-wave on the ductus venosus or spontaneous decelerations on the CTG. Whenever anterograde flow in UA was present, biweekly monitoring (fetal Doppler including interrogation of the ductus venosus plus conventional cardiotocography) was planned, and vaginal delivery (in the absence of other contraindications) was recommended after 37 weeks. If the absent end-diastolic UA flow was detected, subsequent follow-up controls were performed every 48–72 h, and elective cesarean section was indicated at 34 weeks. When reverse end-diastolic UA flow or ductus venosus PI > 95th centile was found, hospitalization and daily monitoring were carried out until elective cesarean section at 30 weeks. Whenever a reverse a-wave flow in the ductus venosus or spontaneous decelerations in the cardiotocography were noted, elective cesarean section was indicated.

#### Perinatal Data

Perinatal data included date and reason for delivery and perinatal mortality.

All data were recorded in a database created on the Research Electronic Data Capture (REDCap) tool (24) hosted by the “imas12” research institute.

### Statistical Analysis

The Strengthening the Reporting of Observational Studies in Epidemiology (STROBE) statement was followed for reporting the results (25).

#### Descriptive Statistics

Continuous variables were expressed in mean (SD) or median (interquartile range) when non-normally distributed. Categorical variables were expressed in percentage (%). Univariate comparisons between the cases in which delivery occurred within 7 days after eoPE diagnosis and those that did not were performed using the *t*-test or Mann–Whitney *U*-test for continuous variables and the chi-square or Fisher’s exact test for categorical variables. Two-sided *p* < 0.05 was considered statistically significant. Statistical package STATA, version 14.1 (TX, United States: StataCorp LP) was used for this analysis.

#### Machine-Learning Model Development

First, we developed a predictive model of the need for delivery within 7 days of diagnosis (model D), considering this as the window of the effect of antenatal corticosteroids for fetal maturation (26). Second, we created a model to calculate the risk of developing HELLP syndrome or *abruptio placentae* at any point after eoPE diagnosis (model HA), as these are the most acute and harder to predict complications.

In order to cover different resource availability scenarios, we developed a reduced version of these models using only demographic characteristics of the patient (D1, HA1) and an extended version adding information available at diagnosis of eoPE (D2, HA2). Accordingly, variables were structured into two datasets: those prior to the onset of PE (baseline) and those evaluated at the moment of diagnosis (diagnosis).

Three preprocessing steps were performed. There were very few missing data in the baseline and “at diagnosis” variables and given the pattern they were considered missing at random. However, there were some variables and some patients with a high number of missing values, especially in the follow-up variables, since this depends on the time a patient participates in the clinical study. First, missing values were imputed using the MissForest imputation technique. This is an imputation method suitable for both categorical and numerical data. It performs an iterative imputation by training a Random Forest model followed by a prediction of the missing values in an iterative process (27). This technique was applied for variables with less than 20% missing data and patients with less than 27% missing data in order to avoid synthetic deviation of the statistical distribution. The remaining variables and patients were directly removed from the dataset. To apply the technique, the Iterative Imputer tool of scikit-learn was used with the Random Forest classifier (28). Treatment of missing values is shown as Supplementary Material 1. Subsequently, nominal variables were categorized and represented by one hot vector. Finally, numerical and ordinal nominal variables were normalized using the Min–MaxScaler tool of the scikit-learn library, which scales the data within a 0–1 range.

The available data present a high dimensionality, and some of them may be redundant or uninformative, which can negatively affect the performance of the classification models. Variable selection (feature selection) methods allow us to obtain the most relevant sets of variables, optimizing the development of machine learning models. We have used a genetic algorithm, a heuristic optimization technique that simulates the natural process of evolution and performs a bio-inspired exploration of a large space of solutions to find the best combination of features, something unfeasible with traditional feature selection techniques in high-dimensionality problems. A mono-objective genetic algorithm (PyWinEA python library)^1^ was used since the main interest was minimizing the number of characteristics. During the process, variables are selected based on a series of previously fixed parameters and a fitness function that needs to be optimized. This function is based on a supervised classification model, using a specific evaluation metric (29). In our case, we tried support vector machine (SVM), K-nearest neighbor (KNN) algorithm, Gaussian Naïve Bayes (GNB), and decision tree (DT) models and selected them relying on the F1-score metric.

Finally, once selected, the best advanced models provided by the genetic algorithm (D2, HA2), we created an interface using Streamlit open-source app framework in Python language where we exported the models to generate a calculator that evaluates the risks as a function of the input variables.

## Results

There were 227 women with eoPE of which 7 were excluded for coexistence with congenital anomalies and only 5 due to lack of determination of angiogenesis biomarkers at diagnosis (*n* = 4), or loss of follow-up (*n* = 1). A total of 215 patients were included, among them, 103 (47.9%) required delivery within 7 days of diagnosis. Baseline characteristics of the study population stratified by the need for delivery within 7 days are depicted in Table 1. There were no statistically significant differences among groups, except for a lower percentage of women with an identifiable *a priori* risk of PE in those with early delivery, mostly at the expense of lower pregestational body mass index and maternal age. This resulted in a lower number of women taking low-dose aspirin before 16 weeks in this subgroup (15.5% vs. 33.9%, *p* < 0.01).

**TABLE 1:** Baseline characteristics of the study population stratified by the presence of preeclampsia complications within 7 days of diagnosis.

Characteristics	Overall (*n* = 215)	Delivery within 7 days	
		No (*n* = 112)	Yes (*n* = 103)
Maternal age (years)	33.4 ± 6.4	33.9 ± 6.6	32.8 ± 6.1
Height (cm)	160 ± 9	159 ± 10	161 ± 6
Pre-pregnancy weight (kg)	68.7 ± 14.2	71.4 ± 15.3	65.5 ± 12.2
Pre-pregnancy BMI (kg/m^2^)	26.7 ± 5.4	27.9 ± 5.9	26.2 ± 5.0
Smoking during pregnancy	8 (3.7)	4 (3.6)	4 (3.9)
Race or ethnic group^†^			
White or Caucasian	116 (54.0)	57 (50.9)	59 (57.3)
Hispanic	71 (33.0)	42 (37.5)	29 (28.2)
Asian	2 (0.9)	1 (0.9)	1 (1.0)
Black or African American	9 (4.2)	6 (5.4)	3 (2.9)
Arab/North African	17 (7.9)	6 (5.4)	11 (10.7)
Risk factors for preeclampsia High			
Previous preeclampsia	24 (11.2)	21 (18.8)	13 (12.6)
Chronic hypertension	35 (16.3)	22 (19.6)	13 (12.6)
Pre-pregnancy diabetes	8 (3.7)	4 (3.6)	4 (3.9)
Chronic kidney disease	5 (2.3)	3 (2.7)	2 (1.9)
Thrombophilia	5 (3.1)	4 (3.6)	1 (1.0)
Systemic lupus erythematosus	2 (0.9)	2 (1.8)	0 (0)
Moderate			
Nulliparity	161 (74.9)	83 (74.1)	78 (75.7)
Age ≥ 40 years	31 (14.4)	21 (18.8)	10 (9.7)
Pre-pregnancy BMI ≥ 35 kg/m^2^	16 (7.4)	14 (12.5)	2 (1.9)
Family history of preeclampsia*	10 (4.7)	6 (5.1)	4 (3.8)
≥1 high-risk or 2 moderate-risk factors	80 (37.2)	42 (46.4)	28 (27.2)
Mode of conception			
Spontaneous	189 (87.9)	139 (87.4)	50 (89.3)
Assisted reproduction technique	24 (11.1)	14 (12.5)	10 (9.7)
Oocyte donation	12 (5.6)	10 (8.9)	2 (1.9)
Low-dose aspirin intake (100 mg/day)			
No	154 (71.6)	70 (62.5)	84 (81.6)
Starting at or before 16 weeks	54 (25.1)	38 (33.9)	16 (15.5)
Starting after 16 weeks	7 (3.3)	4 (3.6)	3 (2.9)
Low-dose heparin prophylaxis			
No	209 (97.2)	108 (96.4)	101 (98.1)
Starting at or before 16 weeks	6 (2.8)	4 (3.6)	2 (1.9)
Starting after 16 weeks	0 (0)	0 (0)	0 (0)
Uterine artery PI > 95th centile at 19–22 weeks γ	85/131 (63.1)	48/79 (60.8)	37/52 (71.2)

*Data are mean ± standard deviation or n (%), unless otherwise stated.*

*First-degree relative (mother or sister) with a history of PE.

*^†^Evaluated after the Bonferroni adjustment.*

*γ Measured in cases from our center.*

*BMI, body mass index.*

As shown in Table 2, regarding eoPE diagnosis, the mean (SD) GA at diagnosis was 29.6 (3.1) weeks. In cases that required delivery within 7 days, GA at diagnosis was significantly lower (29.0 vs. 31.0 weeks, *p* < 0.01), and angiogenesis biomarkers were significantly more altered in the overall ratio and its components, evaluated as absolute numbers, MoM values, or with standardized centile cut-offs. Of note, up to 32% of women who required delivery within 7 days had an sFlt-1/PlGF > 655 at diagnosis. Considering the rest of the blood work, platelets were lower and transaminases higher in those with the need to deliver within 7 days, but these differences were not seen when these parameters were dichotomized according to clinically relevant developed cut-offs. There was a higher rate of growth restricted fetuses among women with prompt delivery after eoPE diagnosis (65% vs. 40.2%, *p* = 0.001), with lower EFW, lower middle cerebral artery PI, and higher PI in the umbilical artery, ductus venosus, and maternal uterine arteries.

**TABLE 2:** Diagnosis characteristics of the study population stratified by the presence of preeclampsia complications within 7 days of diagnosis.

Diagnosis	Overall (*n* = 215)	Delivery within 7 days	
		No (*n* = 112)	Yes (*n* = 103)
GA at diagnosis, median (Q1–Q3)	30.0 (27.4–32.3)	31.01 (28.1–32.6)	29.0 (3.3)
sFlt-1/PlGF			
Median (Q1–Q3)	325 (140–550)	203 (97–380)	460 (285–828)
MoM	83 (33–160)	52 (23–121)	125 (64–228)
>655	41 (19.1)	8 (7.1)	33 (32.0)
sFlt-1			
Median (Q1–Q3) MoM	10,939 (7,877–14,985) 7 (5–10)	9,958 (7,356–13,882) 5.6 (4.4–9.1)	13,116 (8,676–18,008) 8.1 (6.1–11.2)
>95th centile	197 (91.6)	97 (86.7)	100 (97.9)
PlGF			
Median (Q1–Q3)	42 (23–71)	55 (34–86)	29 (19–42)
MoM	0.9 (0.05–0.15)	0.12 (0.08–0.20)	0.06 (0.04–0.10)
<5th centile	191 (88.8)	91 (81.3)	100 (97.9)
Blood pressure (mmHg)			
Systolic	148 ± 14	146 ± 13	149 ± 15
Diastolic	93 ± 9	94 ± 9	93 ± 10
Mean	109 ± 11	109 ± 10	111 ± 11
Platelets			
Absolute (10^3)	223 ± 86	234 ± 63	211 ± 105
<100.000	5 (2.3)	0 (0)	5 (4.9)
Creatinine			
Absolute (mg/dL)	0.65 ± 0.22	0.61 ± 0.12	0.69 ± 0.28
>1.1 mg/dL	3 (1.3)	0 (0)	3 (2.9)
AST	22 (17–30)	21 (16–24)	25 (20–36)
ALT	16 (12–28)	16 (12–24)	19 (13–40)
Estimated fetal weight			
median (Q1–Q3)	1,191 (816–1,741)	1,438 (973–103’)	928 (720–1,272)
<10t^h^ centile	110 (51.2)	45 (40.2)	65 (63.1)
<3rd centile	77 (35.8)	29 (25.9)	48 (46.6)
Umbilical artery PI			
Mean	1.44 ± 0.75	1.27 ± 0.43	1.64 ± 0.96
>95th centile	17 (8.3)	9 (8.0)	8 (8.5)
Middle cerebral artery PI			
Mean	1.69 ± 0.40	1.79 ± 0.35	1.57 ± 0.42
<5th centile	53 (23.9)	16 (23.9)	37 (28.6)
Ductus venosus PI			
Mean	0.51 ± 0.20	0.49 ± 0.13	0.51 ± 0.24
>95th centile	9 (23.9)	0 (0)	9 (3.6)
Mean uterine artery PI			
Mean	1.63 ± 0.62	1.50 ± 0.58	1.80 ± 0.65
>95th centile	38 (23.9)	131 (82.4)	50 (89.3)
Fetal growth restriction			
No	103 (47.9)	67 (59.8)	36 (35.0)
Stage I	92 (42.8)	42 (37.5)	50 (48.5)
Stage II	9 (4.2)	3 (3.7)	6 (5.8)
Stage III	8 (3.7)	0 (0)	8 (7.8)
Stage IV	3 (1.4)	0 (0)	3 (2.9)

*Data are mean ± standard deviation or n (%), unless otherwise stated.*

*AST, aspartate aminotransferase; ALT, alanine aminotransferase; GA, gestational age; MoM, multiples of the median; PI, pulsatility index; PlGF, placental growth factor; Q, quartile; sFlt-1, soluble fms-like tyrosine kinase 1.*

Considering outcomes (Table 3), the median GA at delivery was 32.1 weeks, with a median latency time from diagnosis of 8 days. Those cases that gave birth within 7 days mainly had a maternal indication for delivery in comparison to those with longer latency time (78.4% vs. 69.6%, *p* < 0.01). In the latter group, up to 8.9% delivered for causes unrelated to PE or FGR (intrahepatic cholestasis, premature rupture of membranes, and spontaneous onset of labor). There were no statistically significant differences in terms of the development of severe PE between groups. However, those with earlier delivery after eoPE diagnosis had higher rates of intrauterine demise (8.7% vs. 0%) and suffered from more maternal complications (53.4% vs. 23.2%, *p* < 0.001), especially so due to higher rates of HELLP syndrome (21.4% vs. 3.6%, *p* < 0.001) and *abruptio placentae* (17.5% vs. 4.5%, *p* = 0.002).

**TABLE 3:** Maternal and perinatal outcomes stratified by the presence of preeclampsia complications within 7 days of diagnosis.

Perinatal outcome	Overall (*n* = 215)	Delivery within 7 days		
		No (*n* = 112)	Yes (*n* = 103)	*p*
GA at delivery, median (Q1–Q3)	32.1 (29.0–34.1)	33.8 (31.6–35.4)	29.7 (27.4–32.1)	<0.001
Time to delivery in days, median (Q1–Q3)	8 (3–19)	18 (13–28)	3 (1–5)	<0.001
Reason for delivery				
Maternal, related to preeclampsia	158 (73.5)	78 (69.6)	80 (100)	0.004
Fetal, related to FGR	41 (19.1)	19 (17.0)	22 (21.6)	
Reached 37 weeks	5 (2.3)	5 (4.5)	0 (0)	
Other	11 (5.1)	10 (8.9)	1 (1.0)	
Intrauterine demise	9 (4.2)	0 (0)	9 (8.7)	0.001
Severity features				
Any	137 (63.7)	72 (64.3)	65 (63.1)	0.86
Severely elevated blood pressure	116 (54.0)	63 (56.3)	53 (51.5)	0.48
Elevated liver enzymes	40 (18.6)	16 (14.3)	24 (23.3)	0.09
Low platelets	22 (10.2)	3 (2.7)	19 (18.5)	<0.001
Elevated creatinine	9 (4.2)	4 (3.6)	5 (4.9)	0.64
Maternal complications				
Any	81 (37.7)	26 (23.2)	55 (53.4)	<0.001
Refractory hypertension	34 (15.8)	17 (15.2)	17 (16.5)	0.79
HELLP syndrome	26 (12.1)	4 (3.6)	22 (21.4)	<0.001
*Abruptio placentae*	23 (10.7)	5 (4.5)	18 (17.5)	0.002
Oliguria (<500 mL/24 h)	9 (4.2)	4 (3.6)	5 (4.9)	0.64
Pulmonary edema	6 (2.7)	2 (1.8)	4 (3.9)	0.35
Eclampsia	2 (0.9)	0 (0)	2 (1.9)	0.14

*FGR, fetal growth restriction; GA, gestational age; HELLP, hemolysis, elevated liver enzymes, and low platelets.*

**FIGURE 1:**
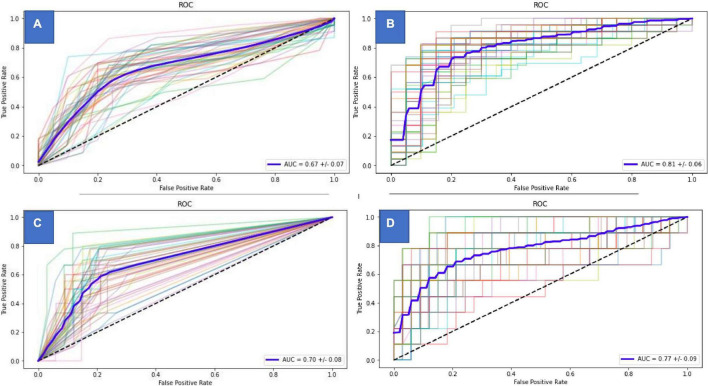
Area under the curve of the different predictive models. **(A)** Delivery within one week predictive model (basal). **(B)** Delivery within one week predictive model (at diagnosis). **(C)** HELLP/Abruptio predictive model (basal). **(D)** HELLP/Abruptio predictive model (at diagnosis).

There were two models developed for both the primary (D) and the secondary (HA) outcomes. The best performance was achieved by the KNN model in time-to-delivery and the TD models for the prediction of HELLP-*abruptio placentae* for the basal approach, and the SVM model performed best in both cases when considering variables at diagnosis of eoPE. The resulting calculator for the prediction of D2 and HA2 can be found in Supplementary Material 2, and the selected variables for each one are in Supplementary Material 3. In the case of model D, the available result is the probability (%) of delivery within 7 days after diagnosis, whereas, in the case of HA, the unbalanced data only allowed for the dichotomic classification of HELLP/*abruptio* risk (yes/no). The performance of the resulting models is depicted in Table 4. Baseline algorithms (D1 and HA1) have an area under the curve (AUC) of 0.68 [95% confidence interval (CI) (0.53–0.82)] in the case of D1 (risk of delivery within 7 days) and of 0.79 (95% CI, 0.66–0.91) in the case of HA1 (risk of developing HELLP syndrome or *abruptio placentae*). These figures are 0.79 (95% CI, 0.66–0.91) and 0.79 (95% CI, 0.55–0.88) for D2 and HA2, respectively, that is, using the available information at diagnosis of eoPE. The AUC using 5 repeats of 10-fold cross-validation of the models is shown in Figure 1.

**TABLE 4:** Diagnostic performance of model D (need to deliver within 7 days of diagnosis) and model HA (occurrence of HELLP syndrome or *abruptio placentae*) in their basic (D1, HA1) and advanced (D2, HA2) versions.

	Area under ROC curve (95% CI)	Sensitivity (95% CI)	Specificity (95% CI)	PPV (95% CI)	NPV (95% CI)
D1	0.68 (0.53–0.82)	63.6 (40.7–82.8)	71.4 (47.8–88.7)	70.0 (45.7–88.1)	65.2 (42.7–83.6)
D2	0.79 (0.66–0.91)	77.3 (54.6–92.2)	80.1 (56.3–94.3)	81.5 (58.1–94.6)	76.2 (52.8–91.8)
HA1	0.77 (0.55–0.88)	60.4 (26.2–87.4)	80.8 (64.5–93.0)	50.0 (21.1–78.9)	87.9 (70.2–96.4)
HA2	0.79 (0.59–0.93)	66.7 (29.9–92.5)	82.8 (65.5–93.2)	51.6 (21.1–78.9)	90.3 (74.2–98.0)

*CI, confidence interval; NPV, negative predictive value; PPV, positive predictive value; ROC, receiver operating characteristic.*

## Discussion

### Main Findings

Our study provides two models based on machine-learning techniques to predict the need for delivery within 7 days (model D) and the future occurrence of HELLP syndrome or *abruptio placentae* (model HA). The advanced versions of such models (D2 and HA2), which include data obtained at diagnosis from angiogenic factors and ultrasonographic study of fetal biometry and Doppler parameters, reached the best performance. It is particularly remarkable their high negative predictive value (NPV) of 76.2% (95% CI, 52.8–91.8%) and 90.3% (95% CI, 74.2–98.0%), respectively.

### Interpretation of the Results

The current evident-based management for eoPE is mainly guided by two clinical trials carried out in the 1990s (30, 31). According to them, expectant management should be pursued in the absence of an imminent threat of complications since it improves perinatal outcomes. Even in eoPE with severe features, this recommendation persists until 34 weeks of gestation. However, the natural course of eoPE in undelivered women tends to be a progressive end-organ dysfunction in which both its speed and type of manifestation have been considered virtually unpredictable. This may explain why it is not uncommon for some clinicians to deliver earlier than stated by current guidelines, aiming to reduce the risk of maternal adverse outcomes at the likely expense of incrementing neonatal morbidity. Therefore, choosing to prolong pregnancies in these circumstances requires meticulous maternal-fetal surveillance and the availability of the appropriate resources to resolve any of its associated complications (32). There have been several advances since those studies were carried out. In the last decade, angiogenic biomarkers (sFlt-1/PlGF ratio) have become available. Their relationship with the evolution of eoPE has allowed us to improve our anticipation of the diagnosis and complications (7, 18). The identification and evaluation of FGR, which is associated in more than half of the cases with eoPE, has also improved with the advances in ultrasound (33). Furthermore, the prognosis of preterm newborns under 34 weeks has improved dramatically, as a result of the continuous improvements in neonatal care (34). This has led to questioning whether the axiom of expectant management of eoPE is still valid and safe enough for any mother and fetus (4) or if there is room for an individualized assessment of risk.

There are paradigmatic examples of the use of clinical tools to predict adverse outcomes in other contexts, such as the scoring systems to estimate the mortality risk on admission to an intensive care unit or after the diagnosis of sepsis (35, 36). The main attempt to develop a tool to identify the risk of adverse maternal outcomes in PE was the full preeclampsia integrated estimate of risk (fullPIERS) model that was published in 2011 (8). It was subsequently externally validated in women with eoPE (37), showing good discrimination for the prediction of any adverse maternal outcome within 48 h of admission, with an area under the receiver operating characteristic curve of 0.80 (95% CI, 0.75–0.86). This model has been criticized for being dominated by variables that are themselves indicative of a complication, such as low platelets or elevated creatinine, and only provides a very short-term prediction. Furthermore, it neither incorporates information from angiogenic markers that have shown promising properties to predict complications, such as HELLP syndrome and *abruptio placentae* (7, 38), nor takes into account parameters of fetal wellbeing. On the contrary, our D2 model provides an expanded prediction of 7 days, which allows a greater margin for decision-making, including transfer to a tertiary center or the administration of corticosteroids. The HA2 model is useful for ruling out acute and highly feared complications, such as HELLP syndrome and *abruptio placentae*, which until now have been deemed completely unpredictable. Of note, the machine-learning methodology selected without any prior condition, both the angiogenic markers and feto-maternal Doppler study among the available parameters to compose the D2 and HA2 models.

The high NPV of the D2 and HA2 models is promising to help better select the appropriate candidates for expectant management among pregnant women with eoPE. These models could also help optimize the administration of antenatal corticosteroids and determine the need for hospitalization. The next steps should focus on the use of these models in a randomized trial to compare maternal and perinatal outcomes between a control group with standard care after eoPE diagnosis and an intervention group in which expectant management or planned early delivery is decided after the knowledge of the results of the D2 and HA2 predictive models.

Predicting maternal and perinatal outcomes in PE remains a challenge, and its unpredictability is a source of stress for patients, their families, and clinicians. Although it has been shown that adopting expectant management before 34 weeks is the best policy, it is not without risks, and this may trigger overattentive women may develop some subjective symptoms (headache and blurred vision), and clinicians prompt early delivery recommendations. On the other hand, not recognizing the onset of some severe complications, which may go unnoticed, such as HELLP syndrome and *abruptio placentae*, can be fatal for both the woman and the fetus. Improving the prediction of time-to-delivery and some complications can help reduce stress, optimize the administration of antenatal corticosteroids, and adjust better both the need for hospitalization and the clinical decision-making of when to deliver. The high NPV of the D2 and HA2 models is promising to help better select the appropriate candidates for expectant management among pregnant women with eoPE. The next steps should focus on the use of these models in a randomized trial to compare maternal and perinatal outcomes between a control group with standard care after eoPE diagnosis and an intervention group in which expectant management or planned early delivery is decided after the knowledge of the results of the D2 and HA2 predictive models.

### Strengths and Limitations

The main limitations of our study come from the use of retrospective data, in which clinicians were not blinded to the knowledge of the sFlt-1/PlGF values or Doppler status. Although they were not used to directly indicate delivery in any case, we cannot exclude that women with higher angiogenic imbalance or fetuses with poorer Doppler status were more closely observed and at higher risk of intervention. However, this represents a real-world evidence scenario in the era of angiogenic markers. In fact, their use has spread widely in recent years because among its strengths is that their simple knowledge is an aid to making better clinical decisions and avoiding thereby serious maternal complications (39, 40). The models have been developed from a relatively small sample size and lack external validation which makes us interpret results with caution. Furthermore, the values of the sFlt-1 and PlGF are not completely interchangeable between different laboratory platforms (17), and a sonographer trained in performing a feto-maternal Doppler study might not always be available in the emergency department where the initial care is given to women with eoPE. Finally, given the high proportion of Caucasian and Hispanic in our cohort, these results may not be applicable to different subsets of patients. Nevertheless, in the case of angiogenesis biomarkers, they have shown good validation in other ethnicities (41, 42).

However, several strengths must be noted as well, such as the use of machine-learning technology to develop the models, which limits pre conceptual bias when selecting the variables in the study. Given its single-center character, there was a great uniformity in management throughout the study period, using systematically the determination of angiogenic markers and ultrasound evaluation of fetal biometry and Doppler parameters at diagnosis.

## Conclusion

We have developed, with the aid of machine-learning techniques and using commonly available clinical data, two models that are applicable at the time of eoPE diagnosis. The first model predicts the need to deliver within 7 days and the second one the future occurrence of HELLP syndrome or *abruptio placentae*. Their high NPV of 76 and 90%, respectively, seems promising for future clinical use as an aid to better select the appropriate candidates for expectant management after the diagnosis of eoPE.

## Data Availability Statement

The raw data supporting the conclusions of this article will be made available by the authors, without undue reservation.

## Ethics Statement

The studies involving human participants were reviewed and approved by the Hospital Universitario 12 de Octubre Ethics Committee. Written informed consent for participation was not required for this study in accordance with the national legislation and the institutional requirements.

## Author Contributions

IH and AG contributed to the conception and design of the study. CV and IH collected the data and drafted the manuscript. PD, JA, PA, CV, IH, and AG analyzed and interpreted the data. JA and AG revised the manuscript. All authors contributed to the article and approved the submitted version.

## Conflict of Interest

CV, IH, and AG received payments for lectures from Roche Diagnostics. The remaining authors declare that the research was conducted in the absence of any commercial or financial relationships that could be construed as a potential conflict of interest.

## Publisher’s Note

All claims expressed in this article are solely those of the authors and do not necessarily represent those of their affiliated organizations, or those of the publisher, the editors and the reviewers. Any product that may be evaluated in this article, or claim that may be made by its manufacturer, is not guaranteed or endorsed by the publisher.

## Footnotes

1
https://github.com/FernandoGaGu/pywinEA
