# Supplementary material for: Prediction of Delivery Within 7 Days After Diagnosis of Early Onset Preeclampsia Using Machine-Learning Models
Source: Front Cardiovasc Med. 2022 Jul 1;9:910701. doi: 10.3389/fcvm.2022.910701 (PMC9283699; doi:10.3389/fcvm.2022.910701)
Supplement: Supplementary file 1 [file Data_Sheet_1.docx]

**Supplementary material 1**. Obtained results for each of the created models to evaluate how the variables with imputed values contribute to the original model

**Table S1a.** Percentage of missing values in the variables

| **Pre-gestational variables** | | | | | |
| --- | --- | --- | --- | --- | --- |
| **Weight** | **BMI** | **Height** |  | | |
| 1.86% | 1.86% | 1.40% |  |  |  |
| **PE episode variables** | | | | | |
| **Uterine artery PI MoM at first scan** | **sFlt-1MoM** | **PlGF MoM** | **PlGF <5^th^ centile** | **sFlt-1 >95^th^ centile** | **Uterine artery Doppler at inclusion** |
| 10.47% | 9.76% | 9.76% | 9.76% | 9.76% | 7.91% |
| **SFlt-1/PlGF MoM** | **Uterine artery PI centile at first scan** | **Right uterine artery PI at first scan** | **Left uterine artery PI at first scan** | **sFlt-1** | **PlGF** |
| 7.91% | 7.44% | 7.44% | 7.44% | 5.58% | 5.58% |
| **MCA PI <5th centile at 1st scan** | **MCA PI at first scan** | **FGR stage** | **Estimated fetal weight centile at inclusion** | **Estimated fetal weight at inclusion** | **MCA PI centile at first scan** |
| 5.58% | 5.12% | 5.12% | 5.12% | 5.12% | 4.19% |
| **UA PI centile at first scan** | **UA PI at first scan** | **UA PI >95th centile at first scan** | **Systolic BP at inclusion** | **Diastolic BP at inclusion** | **Mean BP at inclusion** |
| 4.19% | 4.19% | 4.19% | 3.72% | 3.72% | 3.72% |
| **Nº of antihypertensives** | **sFlt-1/PlGF** | **SFlt-1/PlGF categorized** | **Newborn sex** | **Gestational age at ultrasound** |  |
| 3.72% | 3.25% | 2.79% | 1.86% | 0.47% |  |
| **Follow-up variables** | | | | | |
| **MCA PI <5^th^ centile at second follow up** | **Gestational age at second follow up** | **Uterine artery PI MoM at first follow up** | **Mean Uterine Artery PI centile at first follow up** | **Left Uterine Artery PI at first follow up** | **RIght Uterine Artery PI at first follow up** |
| 19.53% | 19.53% | 19.53% | 19.53% | 15.35% | 15.35% |
| **Mean Uterine Artery PI at first follow up** | **UA PI >95th centile at first follow up** | **UA PI at first follow up** | **MCA PI <5^th^ centile at first follow up** | **MCA PI at first follow up** | **MCA PI centile at first follow up** |
| 15.35% | 12.56% | 12.56% | 12.09% | 11.63% | 11.63% |
| **sFlt-1 at first follow up** | **PlGF at first follow up** | **sFlt-1/PlGF at first follow up** | **Gestational age at ultrasound at first follow up** | **UA PI >95th centile at first follow up** | |
| 5.11% | 4.66% | 4.65% | 2.79% | 12.56% | |

BMI, body mass index; BP, blood pressure; MCA, middle cerebral artery; FGR, fetal growth restriction; SLE, systemic lupus erythematous; PE, preeclampsia; PI, pulstatility index; UA, umbilical artery

**Table S1b.** Developed Models to evaluate how the variables with imputed values contribute to the original model

| **Model** | **Variables used for testing the model** |
| --- | --- |
| D2 | sFlt-1/PlGF, Nº of antihypertensives, Nº of vaginal deliveries, FGR stage, Family history of PE, BMI >35 Kg/m^2^, Ethnicity, AAS, Uterine artery Doppler at inclusion, Estimated fetal weight at inclusion, gestational age at PE |
| D2(A)  (dataset without EFW🡪5.12% of imputed values) | sFlt-1/PlGF, Nº of antihypertensives, Nº of vaginal deliveries, FGR stage, Family history of PE, BMI >35 Kg/m^2^, Ethnicity, AAS, Uterine artery Doppler at inclusion, gestational age at PE |
| D2(B)  (dataset without UA🡪 7.91% of imputed values) | sFlt-1/PlGF, Nº of antihypertensives, Nº of vaginal deliveries, FGR stage, Family history of PE, BMI >35 Kg/m^2^, Ethnicity, AAS, Estimated fetal weight at inclusion, gestational age at PE |
| D2(C)  (dataset without the sFlt-1/PlGF🡪 5.58% of imputed values) | Nº of antihypertensives, Nº of vaginal deliveries, FGR stage, Family history of PE, BMI >35 Kg/m^2^, Ethnicity, AAS, Uterine artery Doppler at inclusion, Estimated fetal weight at inclusion, gestational age at PE |
| D2(D)  (dataset without all the variables with >5% of imputed values) | Nº of antihypertensives, Nº of vaginal deliveries, FGR stage, Family history of PE, BMI >35 Kg/m^2^, Ethnicity, AAS, gestational age at PE |
| HA2 | Gestational age at ultrasound, Estimated fetal weight at inclusion, Prior PE, MCA PI <5th centile at first scan, conception, AAS, Mean BP at inclusion, MCA PI at first scan, BMI >35 Kg/m^2^, FGR stage, Family history of PE, Right uterine artery PI at first scan, SLE, newborn sex, UA PI at first scan, PlGF MoM, sFlt-1/PlGF UA PI >95th centile at first scan, miscarriages, maternal age, Nº of antihypertensives, MCA PI pat first scan, Heparine, chronic hypertension, Uterine artery PI MoMat first scan |
| HA2(A)  (dataset without variables with >9.5% of imputed values) | Gestational age at ultrasound, Estimated fetal weight at inclusion, Prior PE, MCA PI <5th centile at first scan, conception, AAS, Mean BP at inclusion, MCA PI at first scan, BMI >35 Kg/m2, FGR stage, Family history of PE, Right uterine artery PI at first scan, SLE, newborn sex, UA PI at first scan, sFlt-1/PlGF, UA PI >95th centile at first scan, miscarriages, maternal age, Nº of antihypertensives, MCA PI pat first scan, Heparine, chronic hypertension |
| HA2(B)  (dataset without variables with 7-9.5% of imputed values) | Gestational age at ultrasound, Prior PE, conception, AAS, Mean BP at inclusion, BMI >35 Kg/m2, FGR stage, Family history of PE, SLE, newborn sex, UA PI at first scan, PlGF MoM, UA PI >95th centile at first scan, miscarriages, maternal age, Nº of antihypertensives, MCA PI pat first scan, Heparine, chronic hypertension, Uterine artery PI MoM at first scan |
| HA2(C)  (dataset without all the variables with >5% of imputed values) | Gestational age at ultrasound, Prior PE, conception, AAS, Mean BP at inclusion, BMI >35 Kg/m2, FGR stage, Family history of PE, SLE, newborn sex, UA PI at first scan, UA PI >95th centile at first scan, miscarriages, maternal age, Nº of antihypertensives, MCA PI pat first scan, Heparine, chronic hypertension |

AAS, aspirin intake; BP, blood pressure; MCA, middle cerebral artery; FGR, fetal growth restriction; SLE, systemic lupus erythematous; PE, preeclampsia; PI, pulstatility index; UA, umbilical artery

**Table S1c.** Peformance of developed models to test the effect of missing values in the variables

| **Model** | **Accuracy** | **Precision** | **Sensitivity** | **Specificity** | **F1-score** |
| --- | --- | --- | --- | --- | --- |
| **D2** | 0.791 | 0.792 | 0.826 | 0.750 | 0.809 |
| **D2(A)** | 0.767 | 0.760 | 0.826 | 0.700 | 0.792 |
| **D2(B)** | 0.767 | 0.783 | 0.783 | 0.750 | 0.783 |
| **D2(C)** | 0.791 | 0.818 | 0.782 | 0.800 | 0.800 |
| **D2(D)** | 0.791 | 0.850 | 0.739 | 0.850 | 0.791 |
|  |  |  |  |  |  |
| **HA2** | 0.814 | 0.545 | 0.667 | 0.853 | 0.600 |
| **HA2(A)** | 0.744 | 0.417 | 0.556 | 0.794 | 0.476 |
| **HA2(B)** | 0.651 | 0.313 | 0.556 | 0.676 | 0.400 |
| **HA2(C)** | 0.674 | 0.353 | 0.667 | 0.676 | 0.462 |

**Supplementary material 2**. Calculators for the prediction of the need to deliver within 7 days and the occurrence of HELLP syndrome or *abruptio placentae* at the time of diagnosis of early-onset preeclampsia.

Link to prediction of need of delivery within 7 days model:

<https://github.com/Pau29-eng/Temporal_Model_App.git>

Link to prediction of HELLP/*Abruptio placentae* model:

<https://github.com/Pau29-eng/HELLP_Abruptio_Model_App.git>

**Supplementary Material 3.** Included variables in the predictive models

| **Model** | **Baseline** | **PE Diagnosis** |
| --- | --- | --- |
| **D** | **Model D1** (only demographic variables) | **Model D2** (demographic and at eoPE diagnosis variables) |
|  | BMI >35 Kg/m2  Pre-pregnancy weight  Ethnicity  Mode of conception  Family history of PE  Prior PE  Pregestational diabetes  Chronic kidney disease  Parity  Aspirin intake before 16 weeks | BMI >35 Kg/m2  Family history of PE  Ethnicity  Prior vaginal deliveries  Aspirin intake before 16 weeks  Gestational age at diagnosis  sFlt-1/PlGF at diagnosis  Mean uterine artery PI at diagnosis  Estimated fetal weight at diagnosis  Fetal growth restriction at diagnosis |
| **HA** | **Model HA1** (only demographic variables) | **Model HA2** (demographic and at eoPE diagnosis variables) |
|  | BMI >35 Kg/m2  Maternal age >40 years old  Family history of PE  Ethnicity  Mode of conception  Height  Parity  Prior miscarriages  Pregestational diabetes  Chronic kidney disease  Aspirin intake | BMI >35 Kg/m2  Maternal age  Family history of PE  Mode of conception  Prior miscarriages  Prior PE  Systemic lupus erythematosus  Chronic hypertension  Aspirin intake  Treatment with low molecular heparin  Gestational age at diagnosis  Mean blood pressure at inclusion  Number of antihypertensive medications  sFlt-1/PlGF at diagnosis  Estimated fetal weight at diagnosis  Middle cerebral artery PI at diagnosis  Umbilical artery PI at diagnosis  Right uterine artery PI at diagnosis  Fetal sex  Fetal growth restriction (stage IV) |

BMI, body mass index; PE, preeclampsia
